# Clinical characteristics of anti-Ro52/TRIM21-positive versus anti-Ro52/TRIM21-negative patients with anti-synthetase syndrome: a retrospective cohort study

**DOI:** 10.1007/s10067-026-08377-4

**Published:** 2026-09-08

**Authors:** Vanessa Oliveira Teixeira, Tania Wyss, Peter Jandus

**Affiliations:** 1 Faculty of Medicine, University of Geneva, Geneva, Switzerland; 2 Biomedical Data Science Facility, University of Geneva, Geneva, Switzerland; 3 SIB Swiss Institute of Bioinformatics, Lausanne, Switzerland; 4 Division of Immunology and Allergy, Geneva University Hospitals and Faculty of Medicine, Rue Gabrielle-Perret-Gentil 4, CH-1211 Geneva, Switzerland

**Keywords:** Antisynthetase syndrome, Autoantibodies, Interstitial, Lung diseases, Myositis, Retrospective studies

## Abstract

**Objectives:**

Anti-Ro52/TRIM21 antibodies have been linked to interstitial lung disease (ILD) severity in anti-synthetase syndrome (ASS), but their broader clinical relevance remains uncertain. We compared clinical, pulmonary, treatment, and outcome characteristics according to Anti-Ro52/TRIM21 status in a Swiss single-center cohort.

**Methods:**

This retrospective cohort included patients diagnosed with ASS followed at Geneva University Hospitals from 2010 to 2024 who met the Connors criteria and had available anti-Ro52 kDa antibody testing. Group comparisons used two-sided Wilcoxon rank-sum or Fisher’s exact tests, and exploratory associations were assessed using univariate Firth logistic regression.

**Results:**

Among 295 screened records, 38 patients were included (16 anti-Ro52/TRIM21-positive patients and 22 anti-Ro52/TRIM21-negative); mean follow-up was 6.63 ± 5.34 years. Pulmonary involvement was numerically more frequent in anti-Ro52/TRIM21-positive patients (15/16 [93.8%] vs 17/22 [77.3%]; *p* = 0.37), but the regression association was not statistically significant (OR 3.27, 95% CI 0.66–24.51; *p* = 0.18). Severe infections occurred in 6/16 (37.5%) versus 1/22 (4.5%) patients (*p* = 0.028); the univariate OR was 12.60 (95% CI 1.82–255.43; *p* = 0.027). Baseline DLCO and FVC were similar between the groups (*p* = 0.598 and *p* = 0.824, respectively). Treatment patterns differed descriptively, including greater use of biologic in some treatment lines among anti-Ro52/TRIM21-positive patients.

**Conclusions:**

In this small exploratory cohort, severe infections were more frequent among anti-Ro52/TRIM21-positive patients in unadjusted analyses, whereas pulmonary involvement differed only numerically and baseline pulmonary function was similar. Treatment-related confounding factors and the imprecise infection estimate limit interpretation. Larger multicenter studies with standardized longitudinal pulmonary assessment and appropriately adjusted analyses accounting for treatment exposure are needed.

**Table:** 

**Key Points** • *Pulmonary involvement was numerically more frequent in anti-Ro52/TRIM21-positive patients, but the difference was not statistically significant.* • *Severe infections were more frequent in anti-Ro52/TRIM21-positive patients in unadjusted analyses, but the estimate was imprecise and potentially confounded by treatment exposure.* • *Baseline muscle involvement, DLCO, FVC, and prednisone doses at measured time points were broadly similar between the groups.* • *Differences in steroid-sparing treatment patterns were descriptive but should not be interpreted as evidence of a distinct or more severe disease course.*

## Introduction

Anti-synthetase syndrome (ASS) is a rare systemic autoimmune disease within the spectrum of idiopathic inflammatory myopathies and is defined by autoantibodies directed against aminoacyl-transfer RNA synthetases (ARS) [1, 2]. To date, ten distinct ARS antibodies have been described. Anti-Jo-1 is the most frequent and is detected in approximately 60–80% of cases, whereas other recognized antibodies include anti-PL-7, anti-PL-12, anti-EJ, anti-OJ, anti-Zo, anti-Ks, anti-YRS, and the more recently identified anti-VRS and anti-CARS/Ly antibodies [1–4].

Clinically, ASS is characterized by a variable combination of myositis, arthritis, and interstitial lung disease (ILD), often accompanied by Raynaud’s phenomenon, mechanic’s hands, and fever, as reflected in the Connors criteria [5–7]. However, this classic triad is frequently incomplete at presentation, which can make diagnosis challenging in routine care [5–7]. Among all disease manifestations, ILD is of particular clinical importance because it is a major determinant of morbidity, long-term outcome, and survival [5–8]. Pulmonary involvement ranges from mild or slowly progressive disease to rapidly progressive ILD, and antibody-specific subsets may differ in the relative predominance of pulmonary versus muscular manifestations [7–9]. More recent classification initiatives and cohort studies have further highlighted the marked heterogeneity of ASS and the limitations of relying on ARS specificity alone to define clinically meaningful subgroups [9–13].

In addition to ARS antibodies, many patients with ASS harbor antibodies against Ro/Sjögren syndrome-related antigen A (anti-Ro/SSA) [14]. Two major subtypes have been described, anti-Ro52 kDa/TRIM21 and anti-Ro60 kDa, each directed against a distinct antigen [15]. Anti-Ro52/TRIM21 antibodies are commonly detected in systemic autoimmune diseases such as Sjogren’s syndrome and systemic lupus erythematosus and are also frequently found in inflammatory myopathies [14, 15]. In ASS, anti-Ro52/TRIM21 antibodies have been reported in 58–76% of anti-Jo-1-positive patients [14, 16]. Most patients with ASS and anti-Ro52/TRIM21 positivity do not fulfill classification criteria for Sjogren’s syndrome or another defined connective tissue disease, suggesting that this serological association may identify a distinct subset within ASS [16, 17].

The clinical significance of anti-Ro52/TRIM21 positivity in ASS remains incompletely understood. Several studies have suggested an association with more severe pulmonary involvement, particularly severe or progressive ILD [16, 18–21]. La Corte et al. reported an association between anti-Ro/SSA positivity and more severe ILD in ASS [18], whereas other cohorts linked anti-Ro52 positivity to pulmonary severity, disease course, or treatment response [16, 19–21]. More recently, a systematic review and meta-analysis supported an association between anti-Ro52 antibodies and ILD across autoimmune diseases, as well as with rapidly progressive ILD in inflammatory myopathies [22]. Nevertheless, published findings remain partly inconsistent, and the prognostic value of anti-Ro52/TRIM21 positivity in ASS has not yet been fully clarified [16, 18–22].

Given the rarity and clinical heterogeneity of ASS, additional cohort data are needed to better define the relevance of anti-Ro52/TRIM21 status. We therefore aimed to describe the clinical, biological, pulmonary, and therapeutic characteristics of patients with ASS followed at the Division of Immunology and Allergy at Geneva University Hospitals and to explore differences according to anti-Ro52/TRIM21 status. To our knowledge, this is the first Swiss single-center study specifically focused on this question.

## Materials and methods

### Study design, eligibility, and diagnostic confirmation

This retrospective single-center cohort included patients diagnosed with anti-synthetase syndrome (ASS) and followed at Geneva University Hospitals (HUG) between 2010 and 2024. Potential cases were identified from institutional records containing anti-aminoacyl tRNA synthetase (ARS) antibody results. Inclusion required: (1) a documented diagnosis of ASS; (2) fulfillment of the Connors criteria on review of the complete clinical record, defined as an anti-ARS antibody together with at least one characteristic clinical feature, such as myositis, interstitial lung disease, arthritis, Raynaud’s phenomenon, fever, or mechanic’s hands [5]; (3) sufficient clinical documentation for data extraction, and (4) available anti-Ro52 antibody testing allowing classification according to anti-Ro52/TRIM21 status. Patients were excluded if the Connors criteria were not met, clinical records were insufficient, or anti-Ro52/TRIM21 testing was unavailable. Patients without anti-Ro52/TRIM21 testing were excluded because exposure-group status could not be assigned, not because of any clinical outcome. ASS diagnoses were confirmed by retrospective review of the complete clinical and serological record. In one patient, a historical ELISA performed more than 12 years earlier showed very weak anti-Jo-1 reactivity, whereas subsequent slot-blot testing did not demonstrate a significant anti-Jo-1 band. The patient was retained on the basis of the documented historical result and a compatible pulmonary phenotype; the lack of confirmation by a second assay and the resulting diagnostic uncertainty are acknowledged.

### Data collection

Clinical data were extracted from the institutional electronic medical record system (Dossier Patient Informatisé, DPI) and compiled into a dedicated dataset for statistical analysis. General demographic variables included sex, date of birth, smoking status, weight, and height. Disease-specific variables comprised serological profiles, age at diagnosis, inflammatory markers such as C-reactive protein (CRP) concentration and erythrocyte sedimentation rate (ESR), and initial clinical manifestations at presentation. In routine practice at HUG, the ARS antibodies most commonly assessed were anti-Jo-1, anti-PL-7, anti-PL-12, anti-EJ, and anti-OJ. Anti-Ro52/TRIM21 testing was performed as part of clinician-directed myositis/connective tissue disease serology and was not mandated by a uniform study protocol; the precise indication for ordering the test was not consistently documented retrospectively.

Muscle involvement was assessed using serum creatine kinase (CK), aldolase, and troponin levels, as well as muscle biopsy findings when available. Because biopsy findings and aldolase, troponin I, and troponin T measurements were available for only a limited number of patients, they were considered descriptive and were not included in the main analyses. Pulmonary involvement was defined as clinician-documented ASS-related interstitial lung disease supported by compatible thoracic imaging findings, preferably high-resolution computed tomography (HRCT), interpreted in the relevant clinical context. Respiratory symptoms or an isolated pulmonary function test (PFT) abnormality were not sufficient on their own to define pulmonary involvement. Pulmonary involvement was recorded at diagnosis and during follow-up. Baseline forced vital capacity (FVC) and diffusing capacity of the lung for carbon monoxide (DLCO) were defined as the measurements closest time to diagnosis and were expressed as percentages of predicted values. HRCT patterns, radiological progression, and longitudinal changes in pulmonary function could not be analyzed systematically because imaging protocols and reporting details were heterogeneous and serial PFT data were inconsistently available.

Therapeutic variables included methylprednisolone pulses at diagnosis, prednisone dose at prespecified follow-up time points, and the use of corticosteroid-sparing immunosuppressive agents. Outcomes recorded during follow-up included survival, malignancies, severe infections requiring hospitalization, and major cardiovascular or thromboembolic events, including stroke, myocardial infarction, deep vein thrombosis (DVT), and pulmonary embolism. Treatment at the exact time of each severe infection and cumulative time-varying immunosuppressive exposure could not be consistently reconstructed for all events.

### Statistical analysis

All statistical analyses were performed using R software (version 4.3.3). Figures were generated using the ggplot2 package (version 3.5.2). Longitudinal treatment trajectories were visualized with a Sankey diagram generated using the ggalluvial package (version 0.5.12), with proportions displayed within each treatment group at each time point to illustrate treatment distributions over time.

For treatment analyses, individual drugs were grouped into predefined treatment classes. Antimetabolites, abbreviated as AM, included mycophenolate mofetil (MMF), mycophenolic acid (MPA), azathioprine (AZA), mercaptopurine (6-MP) and methotrexate (MTX). Calcineurin inhibitors included tacrolimus (FK506) and ciclosporin (CSA) and were abbreviated as CNI. Biologics included rituximab (RTX), abatacept (ABA), and adalimumab (ADL) and were abbreviated as BIO. Antimalarials included hydroxychloroquine (HCQ) and chloroquine (CQ) and were abbreviated as AMD. Intravenous immunoglobulins (IVIG) were analyzed as a separate treatment class. These treatment classes were used for the grouped treatment plots and Sankey diagram.

Continuous variables were summarized as means ± standard deviations or medians with interquartile ranges, as appropriate. Categorical variables were summarized as counts and percentages. Between-group comparisons of continuous variables, including peak CK, DLCO at diagnosis, FVC at diagnosis, and prednisone dose at each time point, were performed using two-sided Wilcoxon rank-sum tests. Selected categorical comparisons, including pulmonary involvement and severe infection, were performed using two-sided Fisher’s exact tests. Boxplots and violin plots were used to display continuous variable distributions, with the corresponding *p*-values annotated in the figures.

Associations between individual clinical or biological variables and anti-Ro52/TRIM21 status were assessed using Firth’s bias-reduced penalized maximum-likelihood logistic regression, implemented in the logistf package (version 1.26.1). Separate univariate models were fitted for each categorical and continuous variable of interest. Odds ratios (ORs), 95% confidence intervals (CIs), and corresponding *p*-values were extracted for each predictor. Continuous variables were analyzed without categorization and were scaled, when appropriate, to provide clinically interpretable ORs. Given the cohort size and the small number of patients with severe infections, multivariable adjustment was not considered statistically reliable; all regression results should therefore be regarded as exploratory and unadjusted.

A two-sided *p*-value < 0.05 was considered statistically significant. No adjustment for multiple comparisons was applied in this study.

## Ethics approval

This study was approved by the Geneva Cantonal Ethics Committee (Project ID 2023–02346).

## Results

### Patient selection

**Fig. 1:**
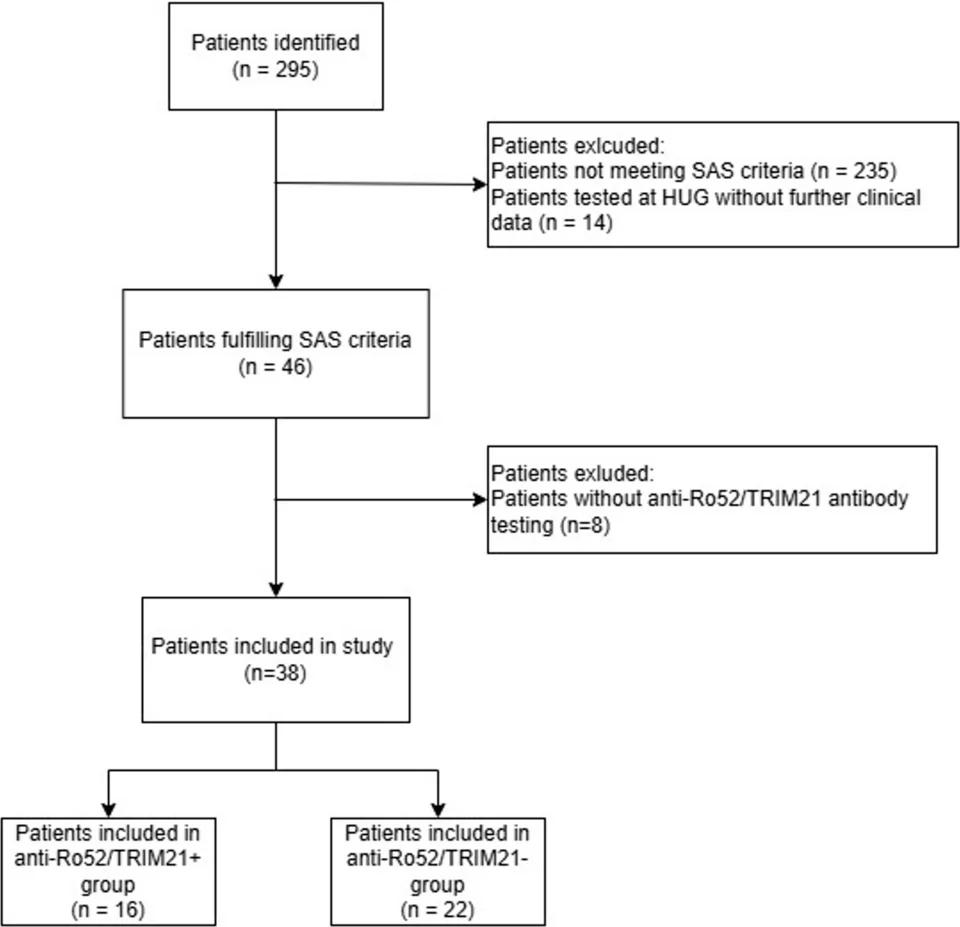
Flowchart of patient selection. Of 295 patient records with anti-aminoacyl-transfer RNA synthetase (*ARS*) antibody results screened, 38 patients fulfilling anti-synthetase syndrome (*ASS*) criteria and with available anti-Ro52/TRIM21 antibody testing were included in the final analysis, comprising 16 anti-Ro52/TRIM21-positive and 22 anti-Ro52/TRIM21-negative patients

A total of 295 patient records with anti-aminoacyl-transfer RNA synthetase (ARS) antibody results were screened. Of these, 235 were excluded because they did not fulfill the criteria for anti-synthetase syndrome (ASS), and 14 were excluded because of insufficient clinical records. Among the remaining 46 patients who fulfilled the ASS criteria, 8 (17.4%) were excluded because anti-Ro52/TRIM21 testing was unavailable. Consequently, 38 patients were included in the final analysis, including 16 anti-Ro52/TRIM21-positive and 22 anti-Ro52/TRIM21-negative patients (Fig. 1).

### Baseline characteristics and follow-up

Baseline demographic, serological, and clinical characteristics are summarized in Table 1. The mean follow-up duration was 6.63 ± 5.34 years. At the data cutoff, 14 patients were no longer under active follow-up at HUG, with 7 patients from each anti-Ro52/TRIM21 group. These 14 patients included 3 who had died, 2 who had relocated outside Switzerland, 7 transfers to private healthcare providers, and 2 for whom the reason was unknown. Excluding deaths, 11 of 38 patients (28.9%) were lost to institutional follow-up.

**Table 1:** Baseline clinical and biological characteristics of patients with anti-synthetase syndrome according to anti-Ro52/TRIM21 status

	Anti-Ro52/TRIM21 + (*n* = 16)	Anti-Ro52/TRIM21 − (*n* = 22)	Total (*n* = 38)
Female sex, *n* (%)	11 (68.8)	18 (81.8)	29 (76.3)
BMI in kg/m^2^ (mean ± SD)	24.9 (± 3.9)	27.4 (± 5.8)	26.3 (± 5.2)
Age at diagnosis, years (mean ± SD)	58.25 (± 14.8)	56.04 (± 10.3)	56.97 (± 13.3)
Tobacco consumption in pack-years (mean ± SD)	4.33 (± 9.5)	16.28 (± 44.2)	11.3 (± 34.5)
Autoantibody profile, *n* (%)			
Anti-Jo-1	9 (56.3)	17 (77.3)	26 (68.4)
Anti-PL-7	3 (18.8)	3 (13.6)	6 (15.8)
Anti-PL-12	1 (6.3)	1 (4.5)	2 (5.3)
Anti-EJ	2 (12.5)	1 (4.5)	3 (7.9)
Anti-OJ	1 (6.3)	0	1 (2.6)
Pulmonary involvement	15 (93.8)	17 (77.3)	32 (84.2)
Pulmonary involvement at diagnosis	14 (87.5)	15 (68.2)	29 (76.3)
Pulmonary involvement developing during follow-up	1 (6.3)	2 (9.1)	3 (7.9)
ILD pattern among patients with pulmonary involvement, *n* (%)			
COP	1 (6.7)	4 (23.6)	5 (15.6)
NSIP	10 (66.7)	10 (58.8)	20 (62.5)
UIP	2 (13.3)	1 (5.9)	3 (9.4)
Other	2 (13.3)	2 (11.8)	4 (12.5)
Other manifestations at diagnosis, *n* (%)			
Myositis	10 (62.5)	13 (59.1)	23 (60.5)
Raynaud’s phenomenon	4 (25.0)	4 (18.2)	8 (21.1)
Mechanic’s hands	6 (37.5)	2 (9.1)	8 (21.1)
Arthritis	8 (50.0)	14 (63.6)	22 (57.9)
Fever	3 (18.8)	2 (9.1)	5 (13.2)
Inflammatory syndrome^†^	14 (87.5)	17 (77.3)	31 (81.6)
Clinical outcomes during follow-up, *n* (%)			
Disease-related death	1 (6.3)	0	1 (2.6)
Associated cancer	2 (12.5)	1 (4.5)	3 (7.9)
Severe infection	6 (37.5)	1 (4.5)	7 (18.4)
Myocardial infarction	1 (6.3)	2 (9.1)	3 (7.9)
Stroke	0	2 (9.1)	2 (5.3)
Pulmonary embolism	2 (12.5)	1 (4.5)	3 (7.9)
Deep vein thrombosis	1 (6.3)	3 (13.6)	4 (10.5)
Follow-up, years, mean ± SD	5.7 ± 5.7	7.32 ± 5.1	6.63 ± 5.3
No longer under active follow-up at HUG at data cutoff, *n* (%)	7 (43.8)	7 (31.8)	14 (36.8)

*ASS* anti-synthetase syndrome, *BMI* body mass index, *COP* Cryptogenic organizing pneumonia, *HUG* Geneva University Hospitals, *ILD* interstitial lung disease, *NSIP* nonspecific interstitial pneumonia, *UIP* usual interstitial pneumonia

^†^Inflammatory syndrome was defined by an elevated *ESR* erythrocyte sedimentation rate and/or *CRP* C-reactive protein concentration

The cohort showed a female predominance, with 29 of 38 patients (76.3%) being women, corresponding to a female-to-male ratio of approximately 3:1. Women accounted for 68.8% (11/16) of the anti-Ro52/TRIM21-positive group and 81.8% (18/22) of the anti-Ro52/TRIM21-negative group. The mean age at diagnosis was 58.3 ± 14.8 years in anti-Ro52/TRIM21-positive patients and 56.0 ± 10.3 years in anti-Ro52/TRIM21-negative patients. The mean body mass index (BMI) was 26.3 ± 5.2 kg/m^2^ overall and was slightly lower in anti-Ro52/TRIM21-positive than in anti-Ro52/TRIM21-negative patients (24.9 ± 3.9 vs 27.4 ± 5.8 kg/m^2^).

A smoking history was reported in 31.6% of patients overall. Cumulative tobacco exposure was lower in the anti-Ro52/TRIM21-positive group than in the anti-Ro52/TRIM21-negative group (4.3 ± 9.5 vs 16.3 ± 44.2 pack-years). Anti-Jo-1 was the most frequently detected ARS antibody, recorded in 26 of 38 patients (68.4%), including 9 of 16 anti-Ro52/TRIM21-positive patients (56.3%) and 17 of 22 anti-Ro52/TRIM21-negative patients (77.3%). In one of these 26 patients, the only evidence of positivity was a historical, very weak ELISA signal that was not confirmed by subsequent slot-blot testing, as detailed in the Methods. Other detected ARS antibodies included anti-PL-7 (6/38 [15.8%]), anti-EJ (3/38 [7.9%]), anti-PL-12 (2/38 [5.3%]), and anti-OJ (1/38 [2.6%]).

### Clinical manifestations at diagnosis

Pulmonary involvement was the most frequent clinical manifestation, affecting 32 of 38 patients (84.2%) overall, and was present at diagnosis in 29 patients (76.3%). It was numerically more frequent in the anti-Ro52/TRIM21-positive group than in the anti-Ro52/TRIM21-negative group (15/16 [93.8%] vs 17/22 [77.3%]); however, this between-group difference was not statistically significant (two-sided Fisher’s exact test, *p* = 0.37).

Myositis occurred at similar frequencies in both groups (62.5% in anti-Ro52/TRIM21-positive patients and 59.1% in anti-Ro52/TRIM21-negative patients), whereas arthritis was more common in anti-Ro52/TRIM21-negative patients (63.6% vs 50.0%). Mechanic’s hands were more frequent in anti-Ro52/TRIM21-positive than in anti-Ro52/TRIM21-negative patients (37.5% vs 9.1%), whereas Raynaud’s phenomenon occurred at comparable frequencies (25.0% vs 18.2%). Fever at diagnosis was uncommon and was reported in 18.8% of anti-Ro52/TRIM21-positive patients and 9.1% of anti-Ro52/TRIM21-negative patients.

Markers of systemic inflammation (CRP and ESR) were frequently elevated. An inflammatory syndrome was present in 31 of 38 patients (81.6%) overall, with a slightly higher frequency in anti-Ro52/TRIM21-positive than in anti-Ro52/TRIM21-negative patients (87.5% vs 77.3%).

### Muscle and pulmonary involvement according to anti-Ro52/TRIM21 status

**Fig. 2:**
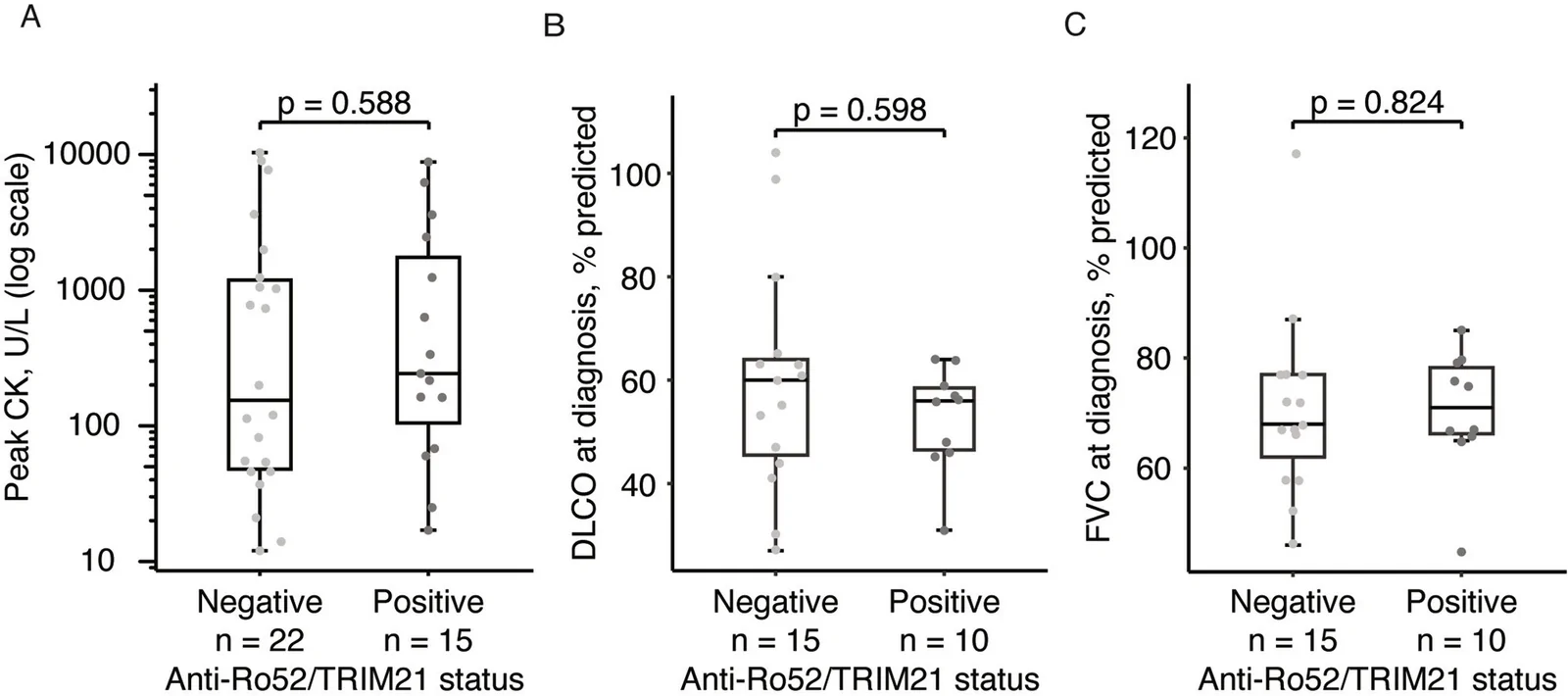
Muscle and pulmonary findings according to anti-Ro52/TRIM21 status. **A** Peak creatine kinase (*CK*) levels in anti-Ro52/TRIM21-negative and anti-Ro52/TRIM21-positive patients. Peak CK measurements were available for 22 of 22 anti-Ro52/TRIM21-negative patients and 15 of 16 anti-Ro52/TRIM21-positive patients. CK values are shown on a logarithmic scale. **B** Baseline diffusing capacity of the lung for carbon monoxide (*DLCO*), expressed as percentages of predicted values. **C** Baseline forced vital capacity (*FVC*), expressed as percentages of predicted values. Baseline DLCO and FVC measurements were available for 15 of 22 anti-Ro52/TRIM21-negative patients and 10 of 16 anti-Ro52/TRIM21-positive patients. Analyses were based on available cases. Dots represent individual patients, boxes indicate the interquartile range, and the central line indicates the median. *p*-values were calculated using two-sided Wilcoxon rank-sum tests to compare distributions between groups

Muscle and pulmonary findings according to anti-Ro52/TRIM21 status are shown in Fig. 2. The median peak creatine kinase (CK) value was numerically higher in anti-Ro52/TRIM21-positive patients than in anti-Ro52/TRIM21-negative patients. However, CK values were widely dispersed in both groups, with broad interquartile ranges and several high outliers, and there was no statistically significant difference between anti-Ro52/TRIM21-positive vs anti-Ro52/TRIM21-negative patients (median 243.0 U/L, IQR 115.0–1851.0 U/L vs 159.5 U/L, IQR 48.9–1191.3 U/L; *p* = 0.588).

Baseline pulmonary function measurements were available for 10 of 16 anti-Ro52/TRIM21-positive patients and 15 of 22 anti-Ro52/TRIM21-negative patients. Median FVC at diagnosis was very similar between the groups (71.0% predicted, IQR 66.3–78.3% predicted vs 68.0% predicted, IQR 62.0–77.0% predicted; *p* = 0.824). Median DLCO was numerically lower in anti-Ro52/TRIM21-positive patients than in anti-Ro52/TRIM21-negative patients (56.0% predicted, IQR 46.5–58.5% predicted vs 60.0% predicted, IQR 45.5–64.0% predicted), but the difference was not statistically significant (*p* = 0.598). These available-case analyses did not show evidence of worse baseline pulmonary functional impairment according to anti-Ro52/TRIM21 status.

### Clinical and biological variables associated with anti-Ro52/TRIM21 positivity

**Fig. 3:**
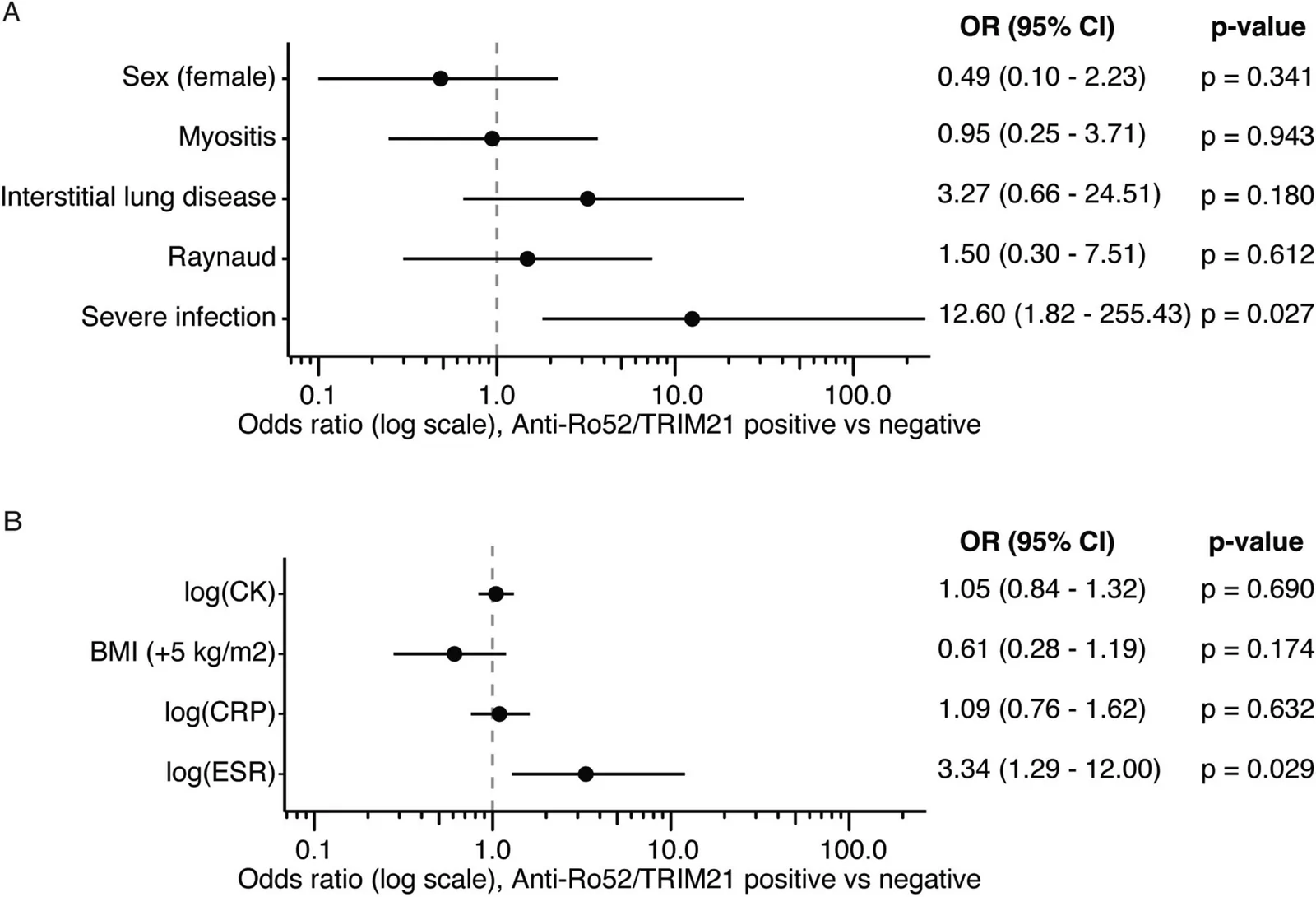
Forest plots showing odds ratios (ORs) with 95% confidence intervals for the associations of individual clinical (**A**) and biological (**B**) variables with anti-Ro52/TRIM21 positivity, as assessed using univariate Firth logistic regression. The vertical dashed line indicates an OR of 1.0. *p*-values are shown for each variable

Associations between individual clinical variables and anti-Ro52/TRIM21 positivity were assessed using univariate Firth logistic regression (Fig. 3A). No significant associations were observed for female sex (OR 0.49, 95% CI 0.10–2.23; *p* = 0.341), myositis (OR 0.95, 95% CI 0.25–3.71; *p* = 0.943), or Raynaud’s phenomenon (OR 1.50, 95% CI 0.30–7.51; *p* = 0.612). Interstitial lung disease showed a positive but non-significant association with anti-Ro52/TRIM21 positivity (OR 3.27, 95% CI 0.66–24.51; *p* = 0.180). Severe infection showed the strongest exploratory association (OR 12.60, 95% CI 1.82–255.43; *p* = 0.027), but this estimate was based on only seven events and had a very wide confidence interval.

Among biological variables (Fig. 3B), higher log-transformed CK and CRP values were associated with numerically increased odds of anti-Ro52/TRIM21 positivity, although neither association reached statistical significance (log_2_[CK], OR 1.05, 95% CI 0.84–1.32; *p* = 0.690; log_2_[CRP + 1], OR 1.09, 95% CI 0.76–1.62; *p* = 0.632). In contrast, higher log_2_-transformed ESR was associated with anti-Ro52/TRIM21 positivity (OR 3.34, 95% CI 1.29–12.00; *p* = 0.029). BMI showed no significant association with anti-Ro52/TRIM21 status (OR 0.61 per 5 kg/m^2^ increase, 95% CI 0.28–1.19; *p* = 0.174). These univariate findings were not adjusted for multiple testing or potential confounding factors.

### Prednisone dose over time according to anti-Ro52/TRIM21 status

**Fig. 4:**
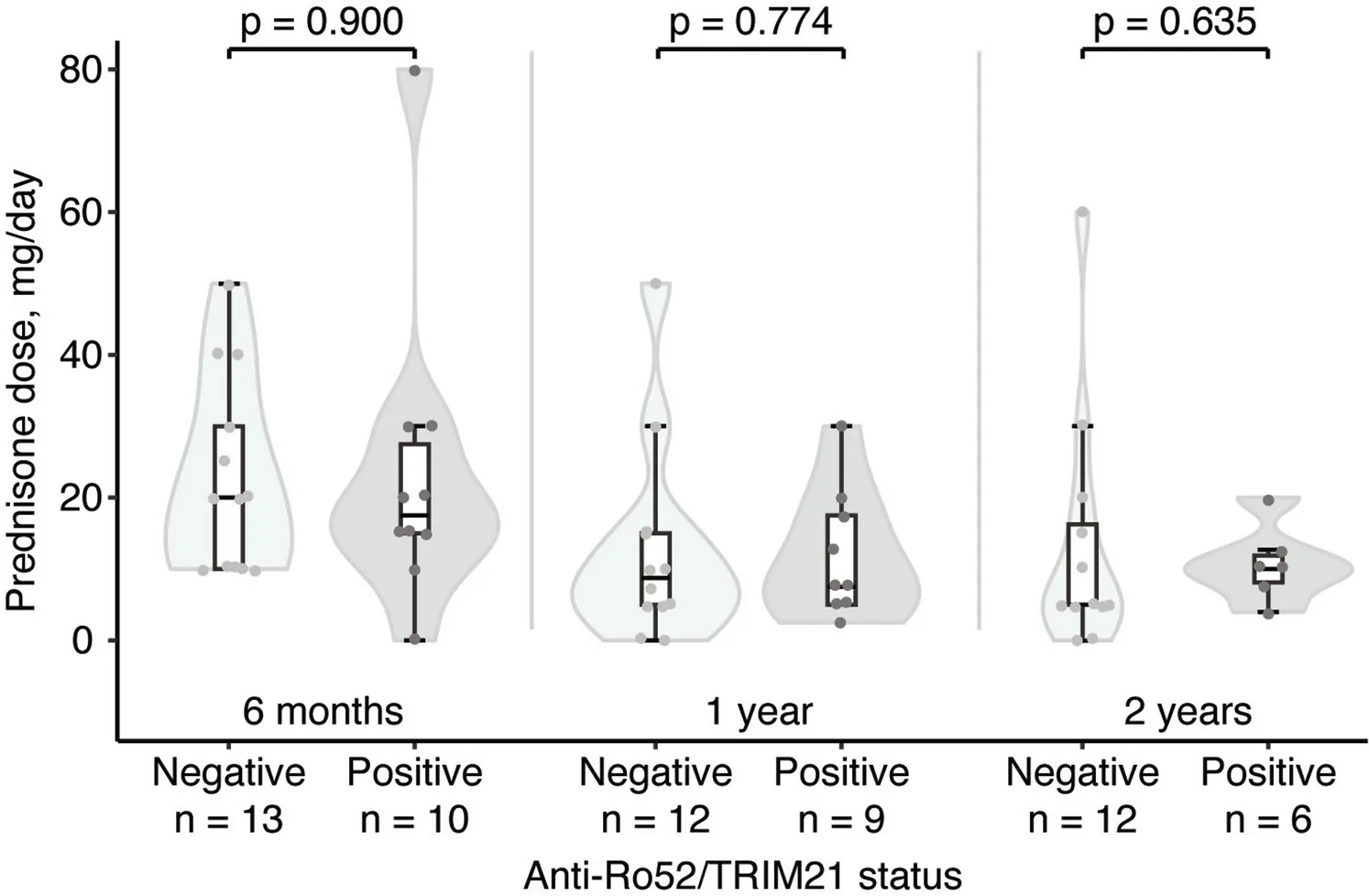
Prednisone dose over time according to anti-Ro52/TRIM21 status. Prednisone doses at 6 months, 1 year, and 2 years after diagnosis are shown for anti-Ro52/TRIM21-negative and anti-Ro52/TRIM21-positive patients. Dots represent individual patients, boxes indicate the interquartile range, and the central line indicates the median. *p*-values were calculated using two-sided Wilcoxon rank-sum tests to determine between-group differences in the median at each time point

Prednisone doses at 6 months, 1 year, and 2 years after diagnosis are shown in Fig. 4. In both groups, prednisone doses decreased over time. No statistically significant differences were observed between anti-Ro52/TRIM21-positive and anti-Ro52/TRIM21-negative patients at 6 months (*p* = 0.900), 1 year (*p* = 0.774), or 2 years (*p* = 0.635). Prednisone dose trajectories at these measured time points were therefore comparable between the groups.

### Treatment patterns according to anti-Ro52/TRIM21 status

**Fig. 5:**
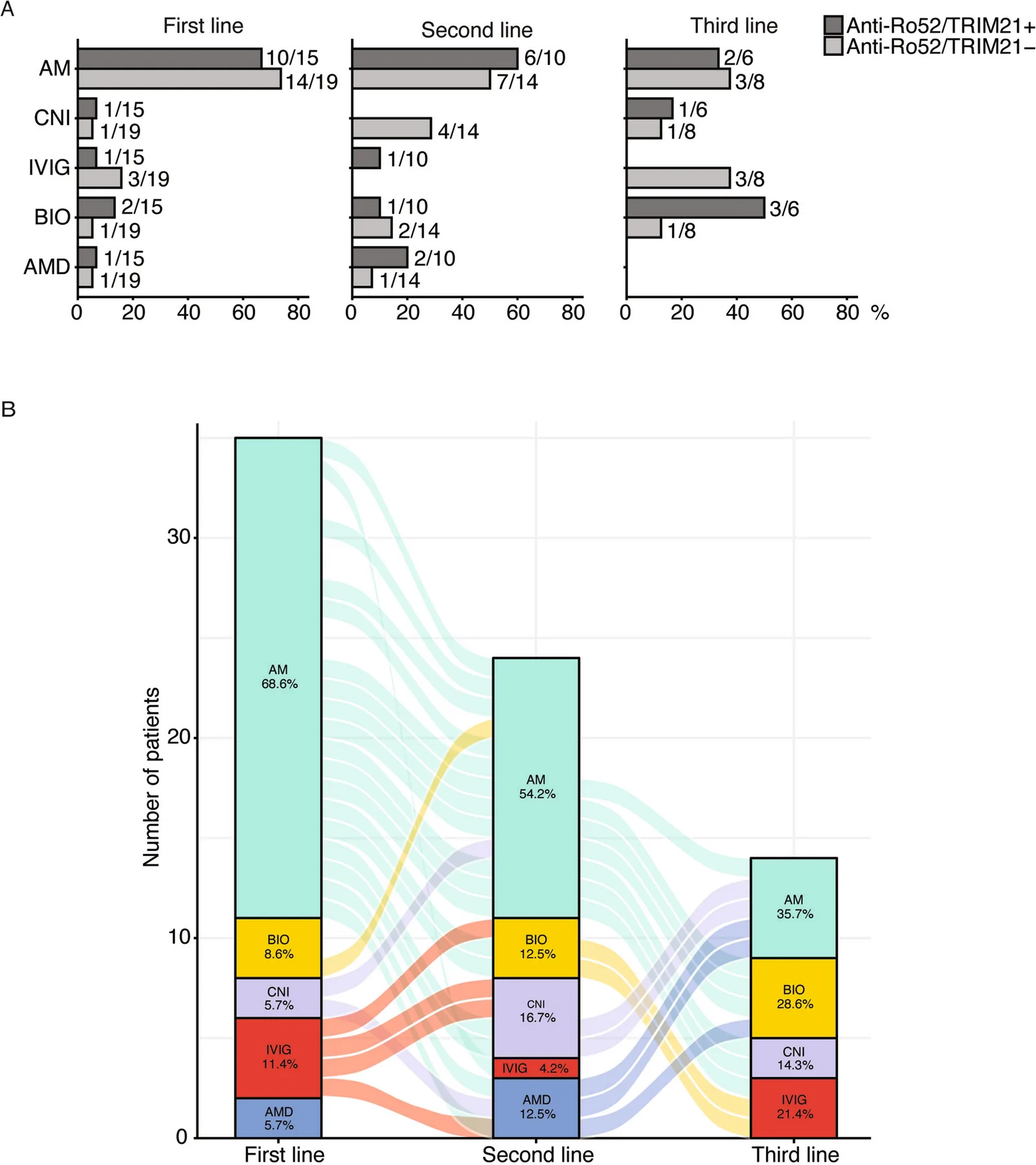
**A** Distribution of treatment classes across first-, second-, and third-line steroid-sparing therapy in anti-Ro52/TRIM21-positive and anti-Ro52/TRIM21-negative patients. Bars represent the proportion of patients receiving each treatment class within the respective treatment line. The ratios next to the bars indicate the number of patients in each treatment class, out of all patients with available treatment information in each anti-Ro52/TRIM21 group. **B** Sankey diagram illustrating treatment trajectories across first-, second-, and third-line steroid-sparing therapies in the overall cohort. The nodes and their heights represent treatment classes and patient counts; percentages are shown within the bars, and flows depict transitions of individual patients between treatment classes across therapy lines. Treatment classes were defined as antimetabolites (mycophenolate mofetil, mycophenolic acid, azathioprine, mercaptopurine, methotrexate; AM), calcineurin inhibitors (tacrolimus, ciclosporin; CNI), intravenous immunoglobulins (IVIG), biologics (rituximab, abatacept, adalimumab; BIO), and antimalarials (hydroxychloroquine, chloroquine; AMD). One patient received concomitant ciclosporin and IVIG as first-line therapy; this patient was counted in both treatment categories in panel A and is displayed as receiving split first-line therapy in panel B

Treatment patterns across first-, second-, and third-line steroid-sparing therapies are shown in Fig. 5A and B. Antimetabolites were the most frequently used treatment class, particularly as first-line therapy, in both anti-Ro52/TRIM21-positive and anti-Ro52/TRIM21-negative patients. Biologics were used more often as first-line therapy in anti-Ro52/TRIM21-positive patients, whereas anti-Ro52/TRIM21-negative patients more frequently received conventional immunosuppressive agents. These comparisons are descriptive and were not adjusted for indication, disease severity, or treatment duration.

In second-line therapy, antimetabolites remained the most common treatment class in both groups, whereas calcineurin inhibitors were prescribed only to anti-Ro52/TRIM21-negative patients and antimalarials were more frequently used in anti-Ro52/TRIM21-positive patients. Third-line therapy was used in fewer patients; biologics were most frequently used in anti-Ro52/TRIM21-positive patients, whereas anti-Ro52/TRIM21-negative patients more often received antimetabolites or IVIG.

The Sankey diagram shows that antimetabolites formed the backbone of treatment, particularly as first line therapy. Treatment trajectories were heterogeneous, with frequent switching between treatment classes during follow-up. The diagram is descriptive and does not establish that anti-Ro52/TRIM21 status independently influenced treatment selection or intensity.

### Clinical outcomes during follow-up

During follow-up, three patients developed malignancies, corresponding to an overall prevalence of 7.9%. Two malignancies occurred in the anti-Ro52/TRIM21-positive group (leiomyosarcoma and squamous cell carcinoma) and one in the anti-Ro52/TRIM21-negative group (melanoma) (Table 1).

Severe infections occurred in 6 of 16 anti-Ro52/TRIM21-positive patients (37.5%) and 1 of 22 anti-Ro52/TRIM21-negative patients (4.5%) (two-sided Fisher’s exact test *p* = 0.028; Fig. 3A). In the corresponding Firth logistic regression, severe infection showed the strongest exploratory association with anti-Ro52/TRIM21 positivity (OR 12.60, 95% CI 1.82–255.43; *p* = 0.027). The estimate was based on only seven patients who experienced the outcome, and the extremely wide confidence interval indicates substantial imprecision and instability in the estimated effect size. Reported infections included postoperative sepsis, sepsis of unknown origin, nosocomial aspiration pneumonia, pyelonephritis with renal abscess, catheter-related bacteremia, and SARS-CoV-2 pneumonia. Because treatment at the time of infection was not consistently captured, this unadjusted difference should be interpreted cautiously.

Cardiovascular and thromboembolic events were also recorded during follow-up. Myocardial infarction occurred in three patients overall, including one anti-Ro52/TRIM21-positive patient and two anti-Ro52/TRIM21-negative patients. Stroke was reported in two anti-Ro52/TRIM21-negative patients. Pulmonary embolism occurred in three patients overall (two anti-Ro52/TRIM21-positive patients and one anti-Ro52/TRIM21-negative patient), whereas deep vein thrombosis was reported in four patients (one anti-Ro52/TRIM21-positive patient and three anti-Ro52/TRIM21-negative patient) (Table 1).

## Discussion

In this single-center retrospective cohort of patients with anti-synthetase syndrome (ASS), the clearest exploratory difference was a higher frequency of severe infections among anti-Ro52/TRIM21-positive patients in unadjusted analyses. Pulmonary involvement was also numerically more frequent in this group, but neither the categorical comparison nor the association in univariate Firth regression was statistically significant. Muscle involvement, baseline pulmonary function, and prednisone doses at the measured follow-up time points were broadly similar between the groups. Accordingly, the present data do not establish that anti-Ro52/TRIM21 positivity defines an independently more severe or more complex ASS phenotype. These findings should be interpreted in the context of the substantial clinical heterogeneity reported across ASS cohorts [7, 11–13, 16–23].

Pulmonary involvement was common in the overall cohort and numerically more frequent among anti-Ro52/TRIM21-positive patients. However, the between-group difference was not statistically significant (Fisher’s exact test *p* = 0.37), and pulmonary involvement was not significantly associated with anti-Ro52/TRIM21 positivity in univariate Firth regression (OR 3.27, 95% CI 0.66–24.51; *p* = 0.18). Baseline FVC and DLCO did not differ significantly between groups. These results should therefore be interpreted as an exploratory numerical pattern rather than as evidence of greater pulmonary involvement or severity associated with anti-Ro52/TRIM21 positivity. The high overall prevalence of pulmonary involvement is nevertheless consistent with the established importance of ILD in ASS and with previous studies investigating anti-Ro52/TRIM21-associated pulmonary phenotypes [3, 7–11, 14–16, 19, 24, 25]. The present study could not comprehensively assess longitudinal pulmonary outcomes: HRCT patterns were not classified using a standardized framework, and radiological progression, rapidly progressive ILD, treatment-related imaging response, and serial FVC and DLCO trajectories could not be analyzed systematically. Consequently, no conclusions regarding ILD severity, progression, or pulmonary prognosis according to anti-Ro52/TRIM21 status can be drawn from these data. Larger cohorts with standardized radiological evaluation and longitudinal pulmonary function assessment are required [24–28].

Severe infections were more frequent among anti-Ro52/TRIM21-positive patients and represented the strongest exploratory signal in the univariate analysis. Nevertheless, the estimate was imprecise (OR 12.60, 95% CI 1.82–255.43) and was based on only seven patients who experienced the outcome. The point estimate should therefore not be interpreted as a precise measure of effect magnitude. Treatment patterns differed between groups, including greater use of biologics in some treatment lines among anti-Ro52/TRIM21-positive patients. More intensive immunosuppression, combined therapy, cumulative glucocorticoid exposure, pulmonary comorbidity, and other unmeasured factors may account for some or all of the observed differences. Treatment at the exact time of infection and cumulative time-varying exposure were not consistently available, and the small number of patients with infections precluded reliable multivariable adjustment. The infection finding should therefore be considered hypothesis-generating rather than as evidence of an independent effect of anti-Ro52/TRIM21 positivity, because the observed association may be influenced by both disease-related factors and immunosuppressive treatment [16, 18–22, 24–32].

The muscular phenotype appeared similar between the groups. Myositis occurred at comparable frequencies. Although the median peak CK was numerically higher in anti-Ro52/TRIM21-positive patients, the distributions overlapped substantially, and the difference was not statistically significant. These results do not support a clearly distinct muscle phenotype according to anti-Ro52/TRIM21 status in this cohort. More broadly, clinically meaningful ASS subgroups may be better characterized by integrating serology, organ involvement, and treatment course than by antibody status alone [9–13, 23, 24, 32–34].

Prednisone doses decreased over time in both groups, with no significant differences at 6 months, 1 year, or 2 years. These measurements do not suggest a difference in glucocorticoid exposure at the assessed time points. However, they do not capture total cumulative dose, pulse therapy, adherence, or time-varying combined immunosuppression and therefore cannot exclude clinically relevant differences in overall treatment burden. Interpretation of glucocorticoid trajectories should therefore account for the broader steroid-sparing treatment context [35–43].

The observed treatment differences should also be interpreted cautiously. Treatment was not protocolized, and the choice of drug likely reflected indication, organ involvement, comorbidities, prior response, calendar period, and physician preference. The descriptive treatment data characterize real-world practice but cannot demonstrate that anti-Ro52/TRIM21 status itself drove therapeutic escalation or identify a distinct treatment-response phenotype. This caution is particularly relevant because ASS is increasingly recognized as a heterogeneous spectrum in which pulmonary, muscular, inflammatory, and therapeutic features intersect [9–13, 24, 32–34, 40]. Current classification efforts likewise favor more integrative approaches combining serological and clinical dimensions [10–13, 33, 34, 40, 44, 45].

This study has several strengths. It provides detailed clinical, serological, pulmonary, and therapeutic characterization of a rare, well-defined ASS cohort from a tertiary referral center and, to our knowledge, represents the first Swiss single-center study focused on the clinical relevance of anti-Ro52/TRIM21 status in ASS. It also includes longitudinal treatment information, including steroid-sparing treatment patterns and prednisone doses at specific time points. These data add real-world context while remaining exploratory.

Several limitations should be emphasized. First, the retrospective single-center design and tertiary referral setting increase the risk of selection bias and limit generalizability. Of 46 patients meeting ASS criteria, 8 were excluded because anti-Ro52/TRIM21 testing was unavailable. Testing was not governed by a uniform protocol, and its clinical indication was not consistently documented; preferential testing of patients with particular phenotypes or greater disease severity therefore cannot be excluded. Second, the cohort size was small (*n* = 38), including only 16 anti-Ro52/TRIM21-positive patients, and the limited number of outcome events reduced the statistical power and precision of the analyses. This may have contributed to the lack of statistical significance for some clinically relevant differences and resulted in very wide confidence intervals, particularly for severe infections. Third, only univariate regression was feasible; the infection analysis could not be adjusted for biologic use, combined immunosuppression, cumulative glucocorticoid exposure, comorbidity, or time-varying treatment. Fourth, HRCT patterns, ILD severity and progression, and longitudinal PFT changes could not be analyzed systematically because data availability and assessment timing were heterogeneous. Fifth, 11 patients were lost to institutional follow-up, and available-case denominators varied across variables. Sixth, one included patient had only a historical, very weak anti-Jo-1 ELISA result that was not confirmed by subsequent slot-blot testing, introducing diagnostic uncertainty. Finally, no correction for multiple comparisons was applied. All findings should therefore be considered exploratory and hypothesis-generating.

Despite these limitations, the observed difference in severe infections may have clinical relevance as a hypothesis-generating signal. Anti-Ro52/TRIM21 status should not, on the basis of the present data, be considered an independent predictor of infection or used alone to guide treatment decisions. Nevertheless, awareness of a potentially increased infectious burden may encourage careful assessment of modifiable infection risks, particularly when biologic agents or combined immunosuppressive regimens are considered. This may include closer review of cumulative immunosuppressive exposure, comorbidities, pulmonary vulnerability, vaccination status, and the need for monitoring or preventive measures according to established clinical practice. Prospective multicenter studies incorporating time-dependent treatment exposure are required to determine whether anti-Ro52/TRIM21 status provides additional value for infection-risk stratification. Such studies could ultimately support more individualized treatment and monitoring strategies aimed at reducing preventable morbidity in patients with ASS.

In conclusion, in this small retrospective cohort, severe infections were more frequent among anti-Ro52/TRIM21-positive patients in unadjusted analyses, whereas pulmonary involvement was only numerically more frequent and baseline muscle involvement, pulmonary function, and prednisone doses at the assessed time points were broadly similar between the groups. Treatment exposure and other unmeasured factors may have contributed to the infection finding, and the present results do not establish an independent effect of anti-Ro52/TRIM21 positivity. Nevertheless, this hypothesis-generating signal may warrant heightened clinical awareness of infection risk, particularly in patients requiring intensive or combined immunosuppression. Larger multicenter studies incorporating longitudinal pulmonary assessment and time-dependent treatment exposure are needed to determine whether anti-Ro52/TRIM21 status can contribute to individualized risk assessment and preventive care in ASS.
